# Prosthetic Valve Endocarditis—Insights from the NatIonal Danish Endocarditis stUdieS (NIDUS) Registry

**DOI:** 10.3390/diagnostics16091372

**Published:** 2026-04-30

**Authors:** Katra Hadji-Turdeghal, Peter Laursen Graversen, Jacob Eifer Møller, Niels Eske Bruun, Jonas A. Povlsen, Claus Moser, Morten Smerup, Jeppe K. Petersen, Amna Alhakak, Eva Havers-Borgersen, Henning Bundgaard, Kasper Iversen, Lauge Østergaard, Lars Køber, Emil L. Fosbøl

**Affiliations:** 1 Department of Cardiology, Copenhagen University Hospital—Rigshospitalet, 2100 Copenhagen, Denmark; katra.hadji-turdeghal@regionh.dk (K.H.-T.);; 2 Department of Cardiology, Copenhagen University Hospital—Amager and Hvidovre Hospital, 2650 Copenhagen, Denmark; 3 Research Unit of Cardiology, Department of Cardiology, Odense University Hospital, 5000 Odense, Denmark; 4 Faculty of Health Sciences, University of Southern Denmark, 5230 Odense, Denmark; 5 Department of Cardiology, Zealand University Hospital, 4000 Roskilde, Denmark; nbru@regionsjaelland.dk; 6 Department of Clinical Medicine, University of Copenhagen, 2200 Copenhagen, Denmark; kasper.karmark.iversen@regionh.dk; 7 Department of Clinical Medicine, Aalborg University, 9260 Aalborg, Denmark; 8 Department of Cardiology, Aarhus University Hospital, 8200 Aarhus N, Denmark; jonapovl@rm.dk; 9 Department of Clinical Microbiology, Copenhagen University Hospital—Rigshospitalet, 2100 Copenhagen, Denmark; claus.moser@regionh.dk; 10 Department of Immunology and Microbiology, University of Copenhagen, 2200 Copenhagen, Denmark; 11 Department of Cardiothoracic Surgery, Copenhagen University Hospital—Rigshospitalet, 2100 Copenhagen, Denmark; morten.holdgaard.smerup@regionh.dk; 12 Department of Cardiology, Copenhagen University Hospital—Herlev and Gentofte, 2730 Herlev, Denmark

**Keywords:** infective endocarditis, IE, registry, cohort, epidemiology

## Abstract

**Background**: Prosthetic valve endocarditis (PVE) is considered a serious complication of valve replacement, and worse outcomes have been reported for PVE versus native valve endocarditis (NVE). Comprehensive data on clinical presentation and practice patterns are lacking. **Methods**: The Danish NatIonal enDocarditis stUdieS (NIDUS) registry was used to obtain data on all patients with left-sided PVE or NVE from 2016–2021 in Denmark. Patients were classified as having a PVE or NVE according to the modified Duke criteria One-year mortality rates for PVE and NVE were assessed using the reversed Kaplan–Maier estimator and adjusted Cox’s regression models. **Results**: In total, 3017 patients were included, of which 791 (26.2%) had PVE and 2226 (73.8%) had NVE. In the PVE group, the median age was 76 years [IQR 70–82], and 74% were males. In the NVE group, the median age was 73 years [IQR 63–80], and 65% were males. The comorbidity burden was similar between groups. The median length of hospital stay was 44 days [IQR 28–52] for PVE and 34 days [IQR 24–45] for NVE. In the PVE group, 567 (74.8%) had definite IE vs. 1863 (82.5%) in the NVE group. At admission, the proportion of patients who presented with embolism (PVE 10.1% vs. NVE 12.5%) and sepsis (PVE 24.4% vs. NVE 23.2%) were comparable. The most common microbiological etiologies for PVE vs. NVE were *Streptococcus* spp. (30.2% vs. 33.8%), *S. aureus* (21.6% vs. 33.4%), and *Enterococcus* spp. (24.5% vs. 14.3%). Surgery during admission was performed in 23.4% of patients with PVE vs. 20.7% with NVE. The absolute risk of one-year mortality in patients with PVE was 30% [95% CI: 27–33], and for NVE, it was 35% [95% CI 33–37] (*p* = 0.0036), with an adjusted hazard ratio (HR) of 0.79 [95% CI 0.68–0.92], with *p* = 0.002. This difference between groups was mainly driven by those above the age of 70 years. **Conclusions**: This nationwide study showed that patients with PVE were older, had similar comorbidity burden, and had a longer length of hospital stay compared to patients with NVE. The PVE group had a numerically higher prevalence of *Enterococcus* spp. Although PVE is a severe condition, our findings indicate that the associated outcomes are more comparable to or even better than NVE than previously assumed, probably related to the unselected and nationwide nature of these data.

## 1. Introduction

Prosthetic valve endocarditis (PVE) is considered a rare and serious complication of surgical and transcatheter valve replacement [1]. Early studies have estimated PVE to account for up to 5% of all IE cases [2]; however, more recent studies have shown that PVE accounts for a significant proportion of all IE cases—estimated to be 20–30% of IE cases [3,4,5,6]. In-hospital mortality in patients with PVE varies but is estimated to be approximately 20–23% in larger IE registries (*n* = 1194–10,841) [3,5], and one-year mortality for PVE has been estimated to be 27–42%—which generally has been reported higher than cohorts of patients with native valve endocarditis [7,8]. However, previous studies on PVE were primarily reported from tertiary hospitals with evident referral and selection biases limiting the generalizability [3,4,5].

Current European Society of Cardiology (ESC) and American Heart Association (AHA) guidelines recommend surgical intervention in PVE in the presence of major complications, including heart failure, severe valvular dysfunction, or peri-/valvular abscess formation [9,10]. Management of patients with PVE is further complicated by these patients’ high prevalence of comorbidities, the technical difficulty and risk associated with secondary valve surgery, and uncertainty regarding optimal timing and patient selection for surgical intervention. These factors are considered to contribute to the persistently reported higher morbidity and mortality associated with PVE compared to NVE and underscore the need for improved evidence to guide clinical management and optimal risk stratification.

Thus, insights into patient characteristics, microbiological etiological profile, and mortality from unselected, nationwide data are needed for the assessment of potential risk factors in patients with PVE and to identify targets for better prevention and treatment.

## 2. Materials and Methods

### 2.1. Data Sources

The Danish NatIonal enDocarditis stUdieS (NIDUS) registry was used to obtain data on all patients with PVE or NVE between 1 January 2016 and 31 December 2021 in Denmark. Patients were classified as having a PVE or NVE according to the modified Duke criteria and European Society of cardiology (ESC) [1]. The registry contains detailed information on relapse and recurrent IE and events as well as detailed data on baseline characteristics, imaging (including echocardiography and positron-emission tomography/computed tomography (PET/CT) scans), microbial etiology, surgical treatment during hospitalization, antibiotic treatment, focus of infection, details on discharge, and clinical outcomes. The design of the registry and the collected data have been described in detail previously [11,12].

### 2.2. Study Population

We identified all patients diagnosed with left-sided PVE and NVE cases between 2016 and 2021 in the NIDUS registry. For the purpose of this study, we included first-time PVE and first-time NVE in the study period. Data on comorbidities, antibiotic treatment and surgical management was obtained from the NIDUS registry.

### 2.3. Covariates

Microbial etiology was assigned according to the most probable primary causative organism, primarily based on blood culture results. In cases where blood cultures were negative, etiology was established using polymerase chain reaction (PCR) testing of excised cardiac valves or other retrieved foreign material. The identified pathogens were then grouped into the following categories: *Staphylococcus aureus*, *Streptococcus* species (spp.), *Enterococcus* spp., coagulase-negative staphylococci (CoNS), and other microorganisms, including fungi and HACEK organisms (i.e., *Haemophilus* spp., *Aggregatibacter actinomycetemcomitans*, *Cardiobacterium hominis*, *Eikenella corrodens*, and *Kingella kingae*).

### 2.4. Outcomes and Follow-Up

The primary objective was to identify the microbiological distribution between NVE and PVE. The secondary outcome was to investigate one-year mortality. Patients were followed from date of IE until the first recorded event: death or end of study on 31 December 2021. Study outcomes were compared between NVE and PVE.

### 2.5. Statistical Analyses

Categorical variables were summarized as counts and percentages, whereas continuous variables were described using medians and interquartile ranges (IQRs). Differences in mortality between groups were evaluated using the chi-square test. Observations with missing data were excluded when the proportion of missing values was over 5%, and percentages were calculated based on the available data. Missing data were reported only for variables with more than 5% missing values, for which proportions were calculated using the total study population as the denominator. The microbiological distribution in patients with PVE and NVE was illustrated with bar charts.

Cumulative incidence of IE-related outcomes was estimated using the Aalen–Johansen method, accounting for the competing risk of all-cause mortality, with between-group differences assessed using Gray’s test. Kaplan–Meier estimates were applied to determine cumulative all-cause mortality, and group differences were examined using the log-rank test [13].

Multivariable Cox’s proportional hazards regression analyses were conducted to estimate adjusted hazard ratios (HRs) for mortality comparing PVE and NVE. The proportional hazards assumption was assessed for the Cox’s regression models using Schoenfeld residuals, and no significant violations were observed. Results were presented as HRs with 95% confidence intervals (CIs). The models were adjusted for clinically relevant covariates, including sex, age (continuous), microbial etiology, diabetes, definite vs. possible IE, dialysis, surgery during admission, chronic obstructive pulmonary disease (COPD), liver disease, active malignancy and heart failure. Patients with missing data for one or more covariates were excluded from the multivariable analyses using a complete-case approach (*n* = 52).

In sensitivity analyses to test the robustness of our findings, we examined the one-year mortality rates for patients with transcatheter aortic valve replacement (TAVI) vs. non-TAVI, early PVE vs. late-PVE, defined as ≤182 days and >183 days, those with only definite IE both PVE or NVE—excluding possible IE [NVE (*n* = 394), PVE (*n* = 203)] and those <75 years and those ≥75 years—and we examined the one-year mortality for vs. possible IE.

All statistical coding and analyses were performed using the Statistical Analysis System (SAS) Enterprise Guide, version 8.4 (SAS Institute Inc., Cary, NC, USA). A *p*-value < 0.05 was considered as statistically significant.

### 2.6. Ethics

The study follows all national ethical principles regarding register-based studies issued by The Danish National Committee on Health Research Ethics and was approved by the regional ethical committee in the Capital Region of Denmark (R-21025667). Informed consent was waivered, and the Danish Data Protection Agency has approved data acquisition (P-2020-92).

## 3. Results

### 3.1. Study Population—Baseline Characteristics

**Figure 1:**
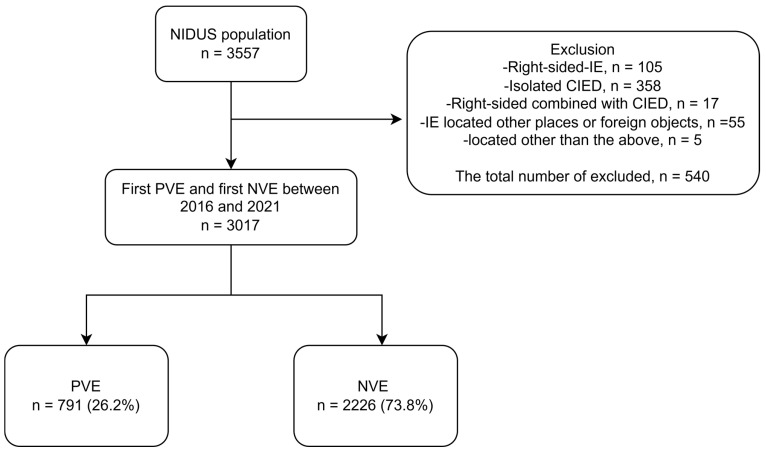
Flowchart of patient selection. NVE = native valve endocarditis. PVE = prosthetic valve endocarditis. CIED = cardiac implantable electronic devices.

In total, 3017 unique patients were included with first-time left-sided IE, of whom 791 (26.2%) had PVE and 2226 (73.8%) had NVE (Figure 1). The median age in patients with PVE was 76.0 years [IQR 68.9–81.5], and 74.0% were males, while in patients with NVE, the median age was 72.9 years [IQR 63.5–80.2], and 64.8% were males. Patients with PVE were hospitalized for a median of 44 days [27–51 days] compared to 33 days [24–45 days] for NVE. In the PVE group, 588 patients (74.3%) had definite IE and 203 (25.7) had possible IE, while in the NVE group, 1832 patients (82.3%) had definite IE and 394 (17.7%) possible IE (Table 1).

### 3.2. PVE and NVE Characteristics and Functional Status

The burden of comorbidities was similar between the two groups for diabetes (PVE 22.3% vs. NVE 23%), COPD (PVE 15% vs. NVE 14.6%), malignant disease (PVE 16.0% vs. NVE 14.4%) and chronic kidney disease (PVE 16.9% vs. NVE 17.3%). However, there were differences between the prevalence of dialysis (PVE 3.7% vs. NVE 5.9%), cardiac implantable electronic devices (CIED) (PVE 22.1% vs. NVE 10.5%), known heart failure (PVE 27.5% vs. NVE 12.0%) and prior IE episodes (PVE 15.8% vs. NVE 1.7%).

In the PVE group, 575 patients (73.6%) were self-reliant in activities of daily living compared to 1529 patients (69.4%) in the NVE group.

### 3.3. Clinical Presentation and Microbiology

At admission, fever (PVE 65.1% vs. NVE 59.7%) and dyspnoea (PVE 36.1% vs. NVE 31.7%) were the most common symptoms. The number of patients who presented with embolism (PVE 10.1% vs. NVE 12.4%) and sepsis at admission (PVE 24.0% vs. NVE 23.0%) were comparable (Table 2). In the PVE and NVE groups, 185 (23.4%) and 460 (20.7%), respectively, underwent surgery during admission (*p* = 0.11).

**Figure 2:**
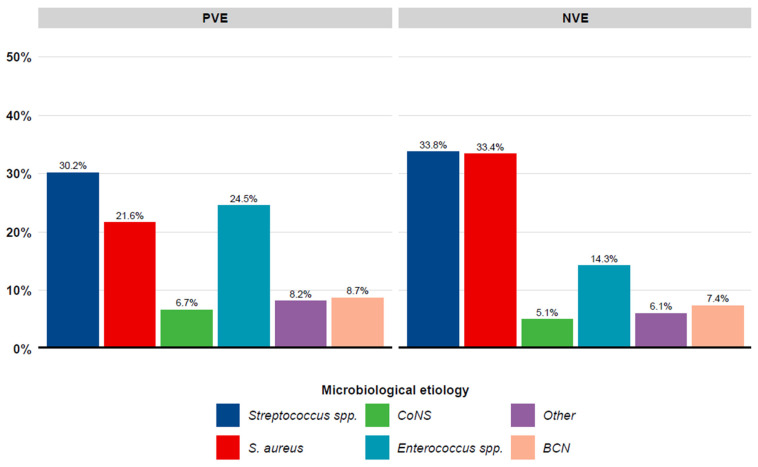
Bar chart of microbiological etiologies stratified on PVE and NVE. BCN = blood culture negative. NVE = native valve endocarditis. PVE = prosthetic valve endocarditis.

The most common microbiological etiologies in the PVE group were *Streptococcus* spp. (30.2%), *S. aureus* (21.6%) and *Enterococcus* spp. (24.5%). The most common microbiological etiologies in the NVE group were *Streptococcus* spp. (33.8%), *S. aureus* (33.4%) and *Enterococcus* spp. (14.3%), (Figure 2).

In the TAVI group, *Streptococcus* spp. (34.8%) and *Enterococcus* spp. (32.9%) were the predominant pathogens, followed by *Staphylococcus aureus* (19.5%), as shown in Supplementary Figure S1.

In the non-TAVI group, *Streptococcus* spp. remained the most common etiology (29.0%), while *S. aureus* (22.2%) and *Enterococcus* spp. (22.3%) contributed similarly, as shown in Supplementary Figure S1.

In early PVE, *Enterococcus* spp. was the most frequent pathogen (30.8%), with higher proportions of coagulase-negative staphylococci (12.8%) and blood-culture-negative cases (16.2%), as shown in Supplementary Figure S2. In late PVE, *Streptococcus* spp. predominated (32.7%), followed by *Enterococcus* spp. (23.5%) and *Staphylococcus aureus* (22.3%), with lower rates of CoNS and BCN, as shown in Supplementary Figure S2.

### 3.4. Echocardiographic (Diagnostic Echocardiography) Findings and PET/CT

Patients with PVE more frequently had aortic valve involvement compared with native valve endocarditis (NVE) (87.5% vs. 58.7%), whereas mitral valve involvement was less common in PVE (22.4% vs. 52.7%). Median vegetation size on the aortic valve was comparable between PVE and NVE patients (9.0 [6.0–13.0] mm vs. 8.0 [5.0–12.0] mm), with similar proportions of vegetations ≥10 mm, and mitral valve vegetation sizes were comparable between groups. Moderate-to-severe valvular regurgitation was more prevalent in NVE for both the aortic (32.3% vs. 10.5%) and mitral valves (38.3% vs. 15.2%). Left-ventricular ejection fraction was lower in PVE (median 55% [45–60]) than in NVE (60% [50–60]), with a higher proportion of patients with PVE exhibiting LVEF < 45% compared to NVE (20% vs. 11%). PET-CT was performed more often in PVE and was more often diagnostic compared to patients with NVE. (26.3% vs. 5.7%) (Table 3).

### 3.5. Mortality

**Figure 3:**
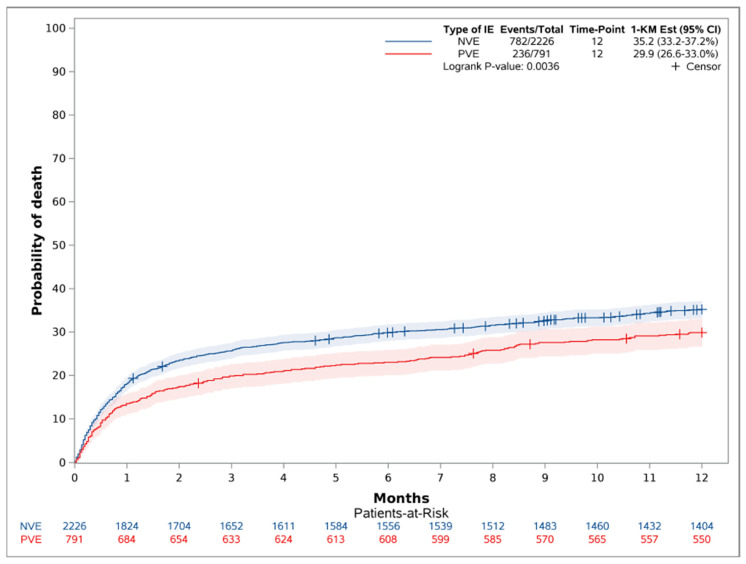
Crude one-year mortality (reversed Kaplan–Meier estimator) stratified on PVE and NVE.

The absolute risk of one-year mortality for those with PVE was 29.9% [95 CI 26.6–33.0], and for NVE, it was 35.2 [33.2–37.2] (*p* = 0.0036), with an adjusted hazard ratio (HR) of 0.79 [95% CI 0.68–0.92], with *p* = 0.002, in favor of patients with PVE (Figure 3).

The one-year mortality for those with TAVI-associated PVE was 32.4%, [95% CI 24.8–39.2], while non-TAVI PVE had a one-year mortality of 29.2% [95% CI 25.6–32.7], with log-rank *p* = 0.010 (Supplementary Figure S3). In addition, early PVE showed a mortality of 19.7% [95% CI 12.2–26.6], whereas late PVE showed a mortality of 31.5% [95% CI 27.9–35.0], with log-rank *p* = 0.0154 (Supplementary Figure S4). 

**Figure 4:**
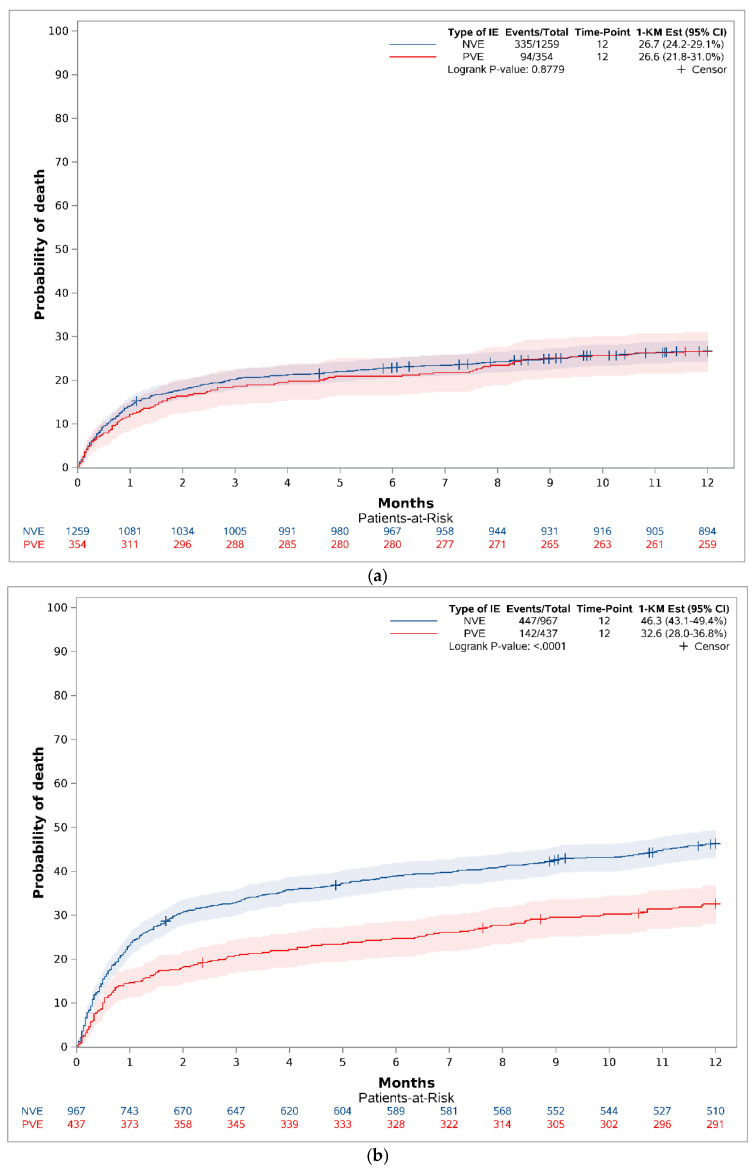
(**a**) Crude one-year mortality (reversed Kaplan–Meier estimator) stratified on PVE and NVE for those <75 years old. (**b**) Crude one-year mortality (reversed Kaplan–Meier estimator) stratified on PVE and NVE for those ≥75 years old.

The one-year mortality rate for those aged <75 years with PVE was 26.6 (95% CI 21.8–31.0) vs. 26.7% (95% CI 24.2–29.1%) for those with NVE, (*p* = 0.8779) (Figure 4a). The one-year mortality rate for those aged ≥75 years with PVE was 32.6 (95% CI 28.0–36.8) vs. 46.3% (95% CI 43.1–49.4%) for those with NVE, (*p* < 0.0001) (Figure 4b).

**Figure 5:**
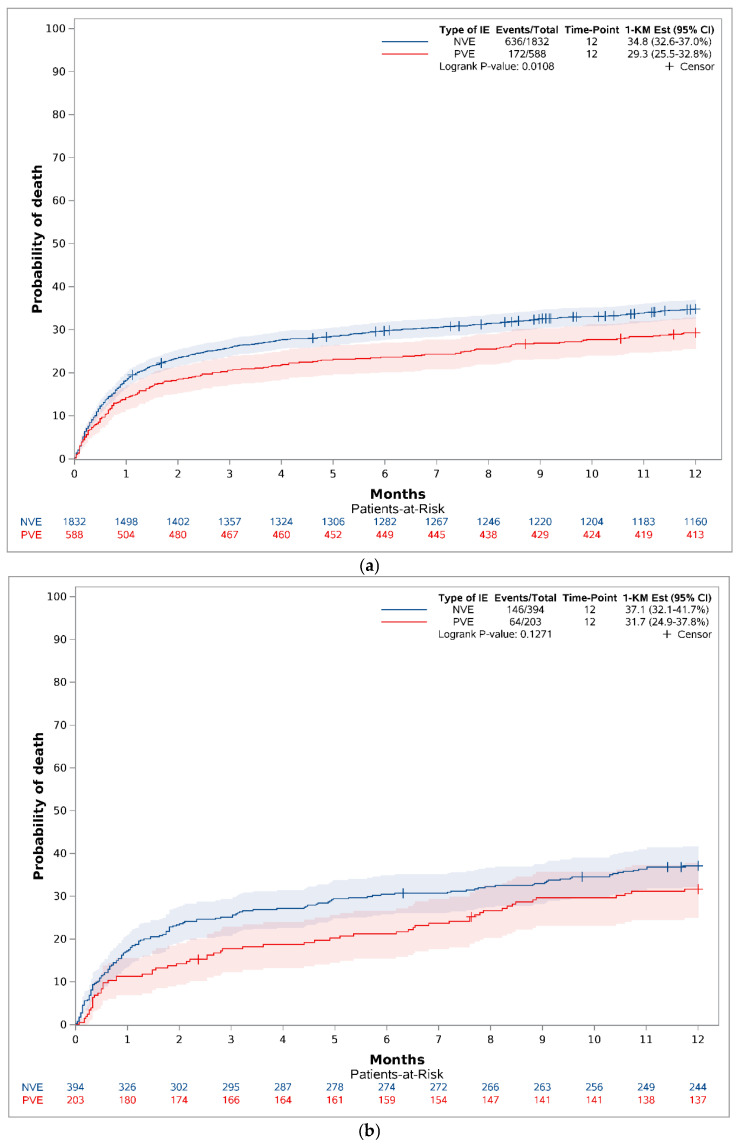
(**a**) Crude one-year mortality (reversed Kaplan–Meier estimator) stratified on PVE and NVE for definite IE. Excluding possible IE in NVE (*n* = 394) and PVE (*n* = 203). (**b**) Crude one-year mortality (reversed Kaplan–Meier estimator) stratified on PVE and NVE for possible IE. NVE = native valve endocarditis. PVE = prosthetic valve endocarditis.

The one-year mortality rate for definite IE for PVE was 29.3 (95% CI 25.5–32.8%), and for NVE, it was 34.8% (95% CI 32.6–37.0%) (*p* = 0.0108) (Figure 5a). The one-year mortality rate for possible IE with PVE was 31.7% (95% CI 24.9–37.8%), and for NVE, it was 37.1 (32.1–41.7%) (*p* = 0.1271) (Figure 5b).

## 4. Discussion

In this nationwide cohort study of patients with first-time left-sided infective endocarditis between 2016 to 2021 in Denmark, we observed distinct differences in microbiological etiology and clinical characteristics between prosthetic valve endocarditis (PVE) and native valve endocarditis (NVE). There were three main findings: First, PVE patients were older, and the IE more often was classified as possible compared to patients with NVE, indicating greater diagnostic uncertainty. Second, the bacterial etiology differed between groups, with more *Enterococcus* spp. in PVE and fewer streptococcal and staphylococcal infections compared with NVE. Third, despite the age difference, mortality was lower in PVE; this is in contrast with other studies and may be explained by diagnostic uncertainty, differences in causative microorganisms, and the inclusion of data from all hospitals rather than only tertiary centers.

This is the first study to assess the microbiological etiological profile and outcomes in patients with PVE from national data. The proportion of patients with PVE out of all IE cases is rising [14]. Reports from large IE registries show an increasing proportion compared with earlier studies on PVE, where the proportion was estimated to be 5% [2]. In our study, PVE accounted for 26%. However, PVE was more frequently classified as possible rather than definite infective endocarditis compared to NVE. This imbalance may introduce classification bias, as the PVE group is likely to include a higher proportion of patients without confirmed infective endocarditis who were nevertheless managed and treated as such out of diagnostic caution.

The proportion of patients with PVE in larger registries has been estimated to be 20–30%, which is in line with our findings [3,4,5,6]. In the ESC-EORP EURO-ENDO registry, PVE comprised 30% (939/3116), possibly reflecting inclusion of repaired valves classified as prostheses and recruitment from tertiary centers [5]. Similar prevalences were reported in GAMES 28% [6] and ICE/ICE-PLUS 24–30% [4], likely due to referral bias and increasing prosthetic valve implantation. In contrast, the Swedish Registry of Infective Endocarditis (SRIE) shows a declining PVE proportion since 2017 [15]. However, as SRIE captures ~85% of all IE cases and excludes patients dying before infectious disease admission, the true prevalence of PVE may be underestimated, as the registry does not fully represent a nationwide population. In smaller IE registries (*n* = 159–1502 included patients), the prevalence of PVE varies substantially from 11–44% [16,17,18,19,20]. The studies are from single centers or centers limited in size with substantial selection bias, thus limiting the generalizability. Nonetheless, similar to larger registries, a higher prevalence of PVE was found compared to early studies on PVE.

The observed microbiological distribution in our study aligns partly with previous reports but also reflects evolving epidemiological patterns in infective endocarditis. In Western countries, *Staphylococcus aureus*, *Streptococcus* spp. and coagulase-negative staphylococci (CoNS), followed by *Enterococcus* species, have historically been reported as the most common causative pathogens in PVE [3,21]. In particular, early PVE—defined as occurring within 60 days of valve implantation—has been characterized by a predominance of *Staphylococcus aureus*, whereas late PVE has more frequently been associated with CoNS and *Enterococcus* species [3]. However, much of this evidence originates from tertiary referral centers, where data completeness varies and selection bias is evident, thereby limiting the generalizability of these findings to unselected national populations. In SRIE, *Streptococcus* spp. (particularly α-hemolytic streptococci) and *Staphylococcus aureus* were the most common pathogens, together accounting for just over half of all cases, while *Enterococcus* spp. accounted for 14% [15]. In our study, in early PVE, *Enterococcus* spp. was the most frequent pathogen (30.8%), with higher proportions of coagulase-negative staphylococci (12.8%) and blood-culture-negative cases (16.2%). In late PVE, *Streptococcus* spp. predominated (32.7%), followed by *Enterococcus* spp. (23.5%) and *Staphylococcus aureus* (22.3%), with lower rates of CoNS and BCN. However, we defined early PVE as ≤6 months and late PVE as >6 months, which could explain some of the differences. The relatively low frequency of CoNS in our study is consistent with Danish nationwide data and likely reflects the unselected population, reducing referral bias toward prosthetic valve cases. Differences in microbiological practices, classification criteria, and temporal or healthcare-related factors may further explain discrepancies with prior studies.

In more contemporary cohorts, increasing healthcare exposure, invasive procedures, and an ageing population appear to have shifted the microbiological spectrum toward *Enterococcus* species and other healthcare-associated pathogens, particularly in PVE. Our nationwide findings support this trend, with *Enterococcus* species accounting for nearly one-quarter of PVE cases. This likely reflects frequent genitourinary or gastrointestinal sources of infection, repeated healthcare contact, and the high burden of comorbidity among patients with prosthetic valves, underscoring the changing epidemiology of PVE [22,23].

Patients with PVE were older and more frequently had known heart failure, cardiac implantable electronic devices, and prior episodes of IE, which is consistent with the existing literature. However, the surgical treatment rates during index hospitalization were similar between groups. This finding may reflect careful patient selection for surgery in PVE, advances in surgical techniques, and improved perioperative management, which may mitigate the traditionally considered poor prognosis associated with prosthetic valve infection.

The finding of lower adjusted mortality in PVE compared with NVE contrasts with older studies reporting worse early outcomes in PVE. However, more recent registry-based studies have suggested that the prognostic gap between PVE and NVE has narrowed, particularly when accounting for age, comorbidities, and microbiological etiology [14,24,25,26]. It is also possible that heightened clinical awareness, earlier use of advanced imaging modalities such as PET/CT, and standardized multidisciplinary endocarditis team management have improved early outcomes in PVE over the past decade, as PET/CT was included in the ESC guidelines [1,27]. In some cases, persistent bacteremia in patients with prosthetic valves can be categorized and managed as PVE, even in the absence of definitive echocardiographic findings due to the poor prognosis of PVE [9].

The main strength of our study is the presentation of nationwide cohort data from unselected consecutive patients with IE. No patients were lost to follow-up due to the unique identification number in Denmark, which allows for linkage to national healthcare registries across regions. However, there are some limitations. First, the observational nature of the study precludes causal inference, and residual confounding cannot be excluded despite multivariable adjustment. Second, although registry data are comprehensive, misclassification of microbiological etiology or clinical variables may occur. Third, information on timing of surgery and antibiotic regimens was not explored in the present analysis and may further influence outcomes. Lastly, classification errors of possible vs. definite IE according to the modified Duke criteria may influence microbiological distribution and outcome estimates; however, this approach reflects real-world clinical practice and is consistent with previous large registry-based studies.

## 5. Conclusions

In conclusion, our nationwide study demonstrates important differences in patient characteristics, diagnostic certainty, and microbiological etiology between PVE and NVE. While PVE remains a condition affecting older patients, one-year mortality appears lower than in NVE, potentially driven by differences in pathogen distribution and advances in diagnostic and therapeutic management but possibly also due to the selection bias in prior studies.

## Figures and Tables

**Table 1:** PVE vs. NVE baseline characteristics.

Characteristic	PVE (*n* = 791) ^1^	NVE (*n* = 2226) ^1^	*p*-Value ^2^
Males	585 (74.0%)	1436 (64.8%)	<0.001
Age	76.0 [68.9–81.5]	72.9 [63.5–80.2]	<0.001
Age in category			
<75 years	354 (44.8%)	1259 (56.6%)	
≥75 years	437 (55.2%)	967 (43.4%)	
Length of hospital stay	44.0 [27.0–51.0]	33.0 [24.0–45.0]	<0.001
Tertiary hospital admission			0.063
No admittance to tertiary hospital	170 (22.9%)	548 (27.6%)	
Admitted at tertiary hospital	573 (77.1%)	1441 (72.4%)	
**Comorbidities**			
Chronic kidney disease	134 (16.9%)	382 (17.3%)	0.8
Dialysis	29 (3.7%)	133 (5.9%)	0.002
Diabetes	176 (22.3%)	510 (23.0%)	0.7
Chronic obstructive pulmonary disease	118 (15.0%)	323 (14.6%)	0.8
Prior malignant disease	126 (16.0%)	319 (14.4%)	0.3
Active malignant disease	55 (7.0%)	218 (9.8%)	0.017
Stroke	164 (20.8%)	287 (12.9%)	<0.001
Known heart failure	217 (27.5%)	266 (12.0%)	<0.001
Native valve disease	69 (8.8%)	340 (15.3%)	<0.001
Congenital heart disease	36 (4.6%)	78 (3.5%)	0.2
Cardiac implantable electronic devices (CIED)	174 (22.1%)	233 (10.5%)	<0.001
Previous heart valve surgery	791 (100.0%)	112 (5.0%)	<0.001
Intravenous drug use			0.008
Never	750 (98.4%)	2070 (97.0%)	
Active	7 (0.9%)	51 (2.4%)	
Previously	5 (0.7%)	14 (0.7%)	
Prior IE episode	124 (15.8%)	37 (1.7%)	<0.001
Duke/2015 ESC diagnostic criteria			
Definite	588(74.3%)	1832 (82.3%)	
Possible	203(25.7%)	394 (17.7%)	<0.001
Valve surgery during admission	185 (23.4%)	460 (20.7%)	0.11
**Behavioral risk factors**			
Smoking status			<0.001
Active	105 (13.9%)	467 (22.2%)	
Previously	326 (43.1%)	747 (35.4%)	
Alcohol_male			0.002
≤14 units per week	374 (80.4%)	863 (74.0%)	
>14 units per week	91 (19.6%)	303 (26.0%)	
Alcohol_female			<0.001
≤7 units	129 (93.5%)	436 (80.9%)	
>7 units	9 (6.5%)	103 (19.1%)	
Self-reliant in activities of daily living	575 (73.6%)	1529 (69.4%)	0.063

^1^ n (%); median [Q1–Q3]. ^2^ Pearson’s chi-squared test; Wilcoxon’s rank sum test; Fisher’s exact test.

**Table 2:** Symptoms at admission.

Characteristic	PVE *N* = 791 ^1^	NVE *N* = 2226 ^1^	*p*-Value ^2^
Acquisition of infection			0.023
Community-acquired	514 (65.5%)	1541 (69.6%)	
Healthcare-associated	180 (22.9%)	484 (21.9%)	
Unknown	91 (11.6%)	189 (8.5%)	
Fever at admission	493 (65.1%)	1276 (59.7%)	0.009
Weight loss at admission >5 kg	100 (13.7%)	314 (15.0%)	0.4
Myalgias located at the proximal musculature	191 (25.5%)	586 (27.7%)	0.2
Dyspnea at admission	272 (36.1%)	674 (31.7%)	0.027
Osler nodes	3 (0.4%)	11 (0.5%)	>0.9
Splinters	37 (4.7%)	116 (5.2%)	0.6
Petechiae	16 (2.0%)	52 (2.3%)	0.6
Janeway lesions	8 (1.0%)	23 (1.0%)	>0.9
Other clinical findings	2 (0.3%)	9 (0.4%)	0.7
Embolization at admission	80 (10.1%)	275 (12.4%)	0.10
Embolization to CNS	57 (7.2%)	198 (8.9%)	0.8
Embolization to liver	0 (0.0%)	3 (0.13%)	>0.9
Embolization to spleen	13 (1.64%)	33 (1.48%)	0.4
Embolization to lungs	2 (0.25%)	13 (0.58%)	0.5
Embolization to extremities or peripheral vasculature	13 (1.64%)	29 (1.30%)	0.2
Embolization to eyes	1 (0.12%)	11 (0.49%)	0.3
Embolization to other organs	7 (0.88%)	33 (1.48%)	0.4
Signs and symptoms of heart failure at admission	23 (2.9%)	54 (2.4%)	0.5
Signs of heart block at admission	58 (7.3%)	86 (3.9%)	<0.001
Signs of severe valve regurgitation at admission	39 (4.9%)	188 (8.4%)	0.001
Sepsis at admission	190 (24.0%)	513 (23.0%)	0.6
Other complications at admission	26 (3.3%)	72 (3.2%)	>0.9

^1^ n (%). ^2^ Pearson’s chi-squared test; Fisher’s exact test.

**Table 3:** Diagnostic echocardiography and PET/CT.

Characteristic	PVE *N* = 791 ^1^	NVE *N* = 2226 ^1^	*p*-Value ^2^
IE on aortic valve	690 (87.5%)	1297 (58.7%)	<0.001
Vegetation size aortic valve [median]	9.0 [6.0–13.0]	8.0 [5.0–12.0]	0.10
Vegetation size aortic valve categories			0.3
Vegetation < 10 mm	395 (76.7%)	853 (74.2%)	
Vegetation ≥ 10 mm	120 (23.3%)	296 (25.8%)	
Aortic valve regurgitation			<0.001
None/Mild	569 (89.5%)	829 (67.7%)	
Moderate	40 (6.3%)	203 (16.6%)	
Severe	27 (4.2%)	192 (15.7%)	
Aortic root abscess			<0.001
No	492 (75.1%)	1085 (87.9%)	
Possible	89 (13.6%)	93 (7.5%)	
Definite	74 (11.3%)	56 (4.5%)	
IE on mitral valve	174 (22.4%)	1165 (52.7%)	<0.001
Vegetation size mitral valve [median]	10.0 [7.0–14.0]	10.0 [8.0–15.0]	0.046
Vegetation size mitral valve categories			0.3
Vegetation < 10 mm	87 (59.2%)	577 (55.0%)	
Vegetation ≥ 10 mm	60 (40.8%)	472 (45.0%)	
Mitral valve regurgitation			<0.001
None/Mild	134 (84.8%)	687 (61.6%)	
Moderate	18 (11.4%)	248 (22.2%)	
Severe	6 (3.8%)	180 (16.1%)	
Mitral valve abscess			0.5
No	152 (96.2%)	985 (93.5%)	
Possible	5 (3.2%)	48 (4.6%)	
Definite	1 (0.6%)	20 (1.9%)	
Left-ventricular ejection fraction [median]	55.0 [45.0–60.0]	60.0 [50.0–60.0]	<0.001
LVEF in categories			<0.001
55–60%	366 (53.4%)	1368 (73.5%)	
45–54%	179 (26.1%)	287 (15.4%)	
30–44%	93 (13.6%)	153 (8.2%)	
<30%	47 (6.9%)	52 (2.8%)	
PET-CT scan performed	569 (72.0%)	1466 (65.9%)	0.002
PET-CT diagnostic (focal uptake valve/heart)	148 (26.3%)	83 (5.7%)	<0.001

^1^ n (%); median [Q1–Q3], ^2^ Pearson’s chi-squared test; Wilcoxon’s rank sum test; Fisher’s exact test.

## Data Availability

The data supporting this article cannot be made publicly available due to General Data Protection Regulation (GDPR) restrictions on personal data. However, it can be accessed upon reasonable request to the corresponding author, provided permission is granted by the institution responsible for the data.

## Supplementary Materials

The following supporting information can be downloaded at: https://www.mdpi.com/article/10.3390/diagnostics16091372/s1, Figure S1: Barchart of the microbiological distribution TAVI PVE vs. non-TAVI PVE. TAVI = transcatheter aortic valve replace-ment. NVE = native valve endocarditis. PVE = prosthetic valve endocarditis. BCN = blood culture negative; Figure S2: Barchart of the microbiological distribution early PVE vs. late-PVE defined as defined as ≤182 days and >183 days. NVE = native valve endocarditis. PVE = prosthetic valve endocarditis. BCN = blood culture negative; Figure S3: Crude one-year mortality (reversed Kaplan-Meier estimator) stratified on TAVI PVE vs. non-TAVI PVE. TAVI= transcatheter aortic valve replacement. NVE = native valve endocarditis. PVE = prosthetic valve endocarditis; Figure S4: Crude one-year mortality (reversed Kaplan-Meier estimator) stratified on early PVE vs. late-PVE defined as defined as ≤182 days and >183 days. NVE = native valve endocarditis. PVE = prosthetic valve endocarditis.
