# Supplementary material for: Prosthetic Valve Endocarditis—Insights from the NatIonal Danish Endocarditis stUdieS (NIDUS) Registry
Source: Diagnostics (Basel). 2026 Apr 30;16(9):1372. doi: 10.3390/diagnostics16091372 (PMC13163577; doi:10.3390/diagnostics16091372)
Supplement: Supplementary file 1 [file diagnostics-16-01372-s001.zip › diagnostics-4193785-supplementary.pdf]

Supplemental figures

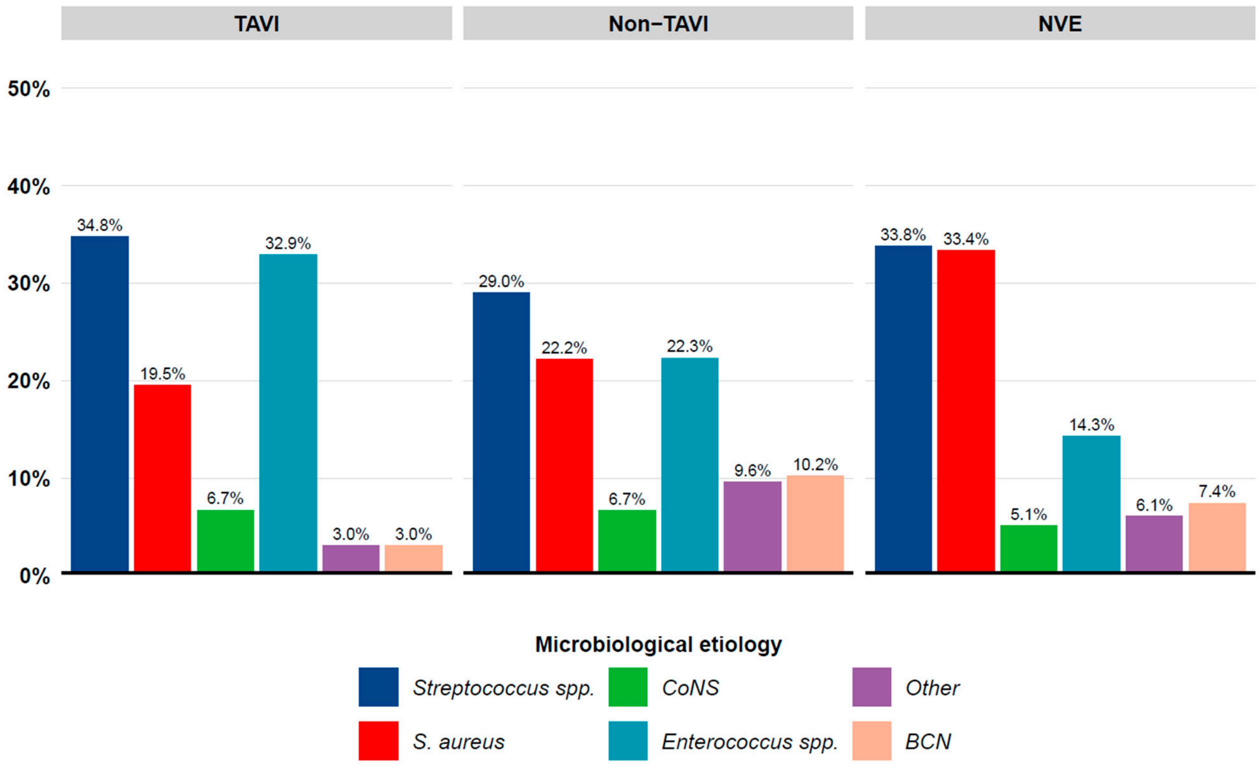

Supplemental Figure S1 Barchart of the microbiological distribution TAVI PVE vs. non-TAVI PVE. TAVI= transcatheter aortic valve replacement. NVE = native valve endocarditis. PVE = prosthetic valve endocarditis. BCN= blood culture negative

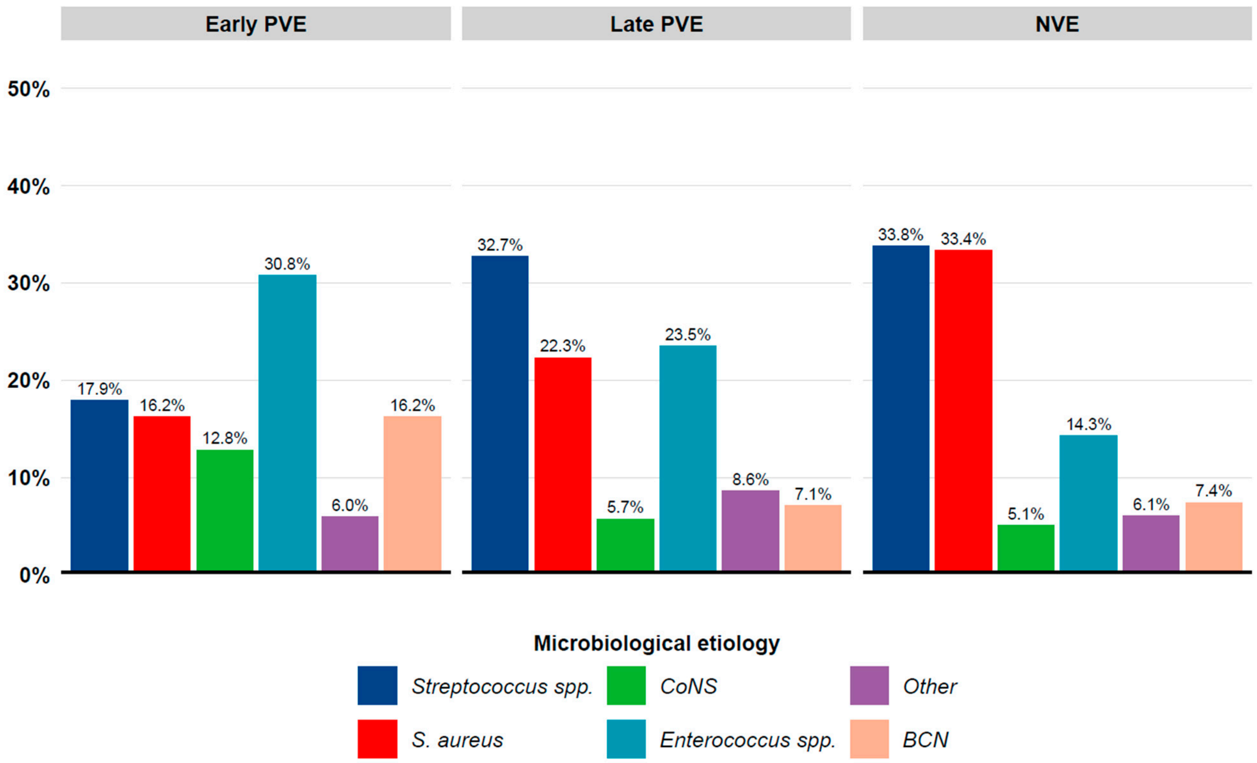

Supplemental Figure S2 Barchart of the microbiological distribution early PVE vs. late-PVE defined as defined as ≤ 182 days and >183 days. NVE = native valve endocarditis. PVE = prosthetic valve endocarditis. BCN= blood culture negative.

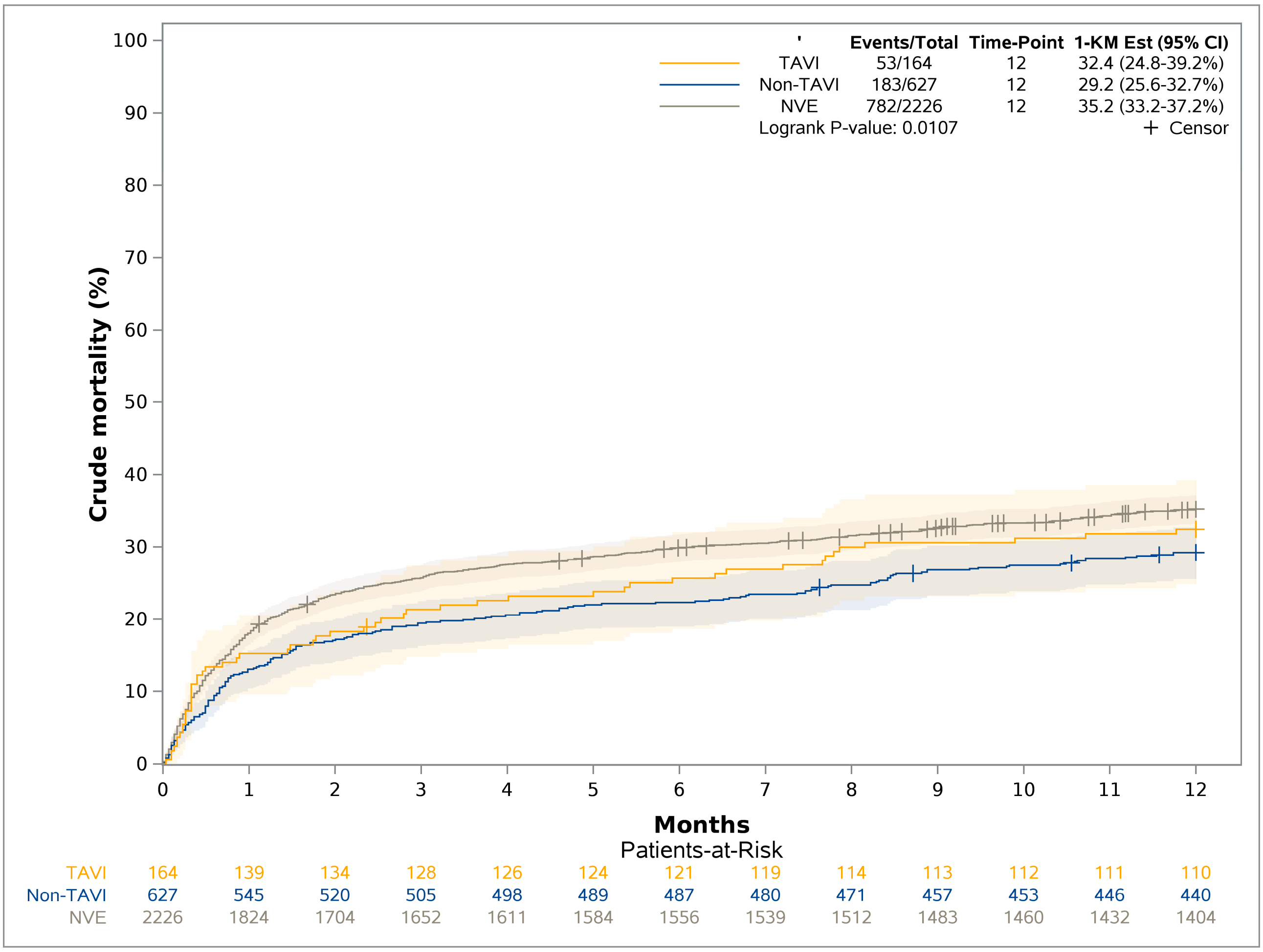

Supplemental Figure S3 Crude one-year mortality (reversed Kaplan-Meier estimator) stratified on TAVI PVE vs. non-TAVI PVE. TAVI= transcatheter aortic valve replacement. NVE = native valve endocarditis. PVE = prosthetic valve endocarditis

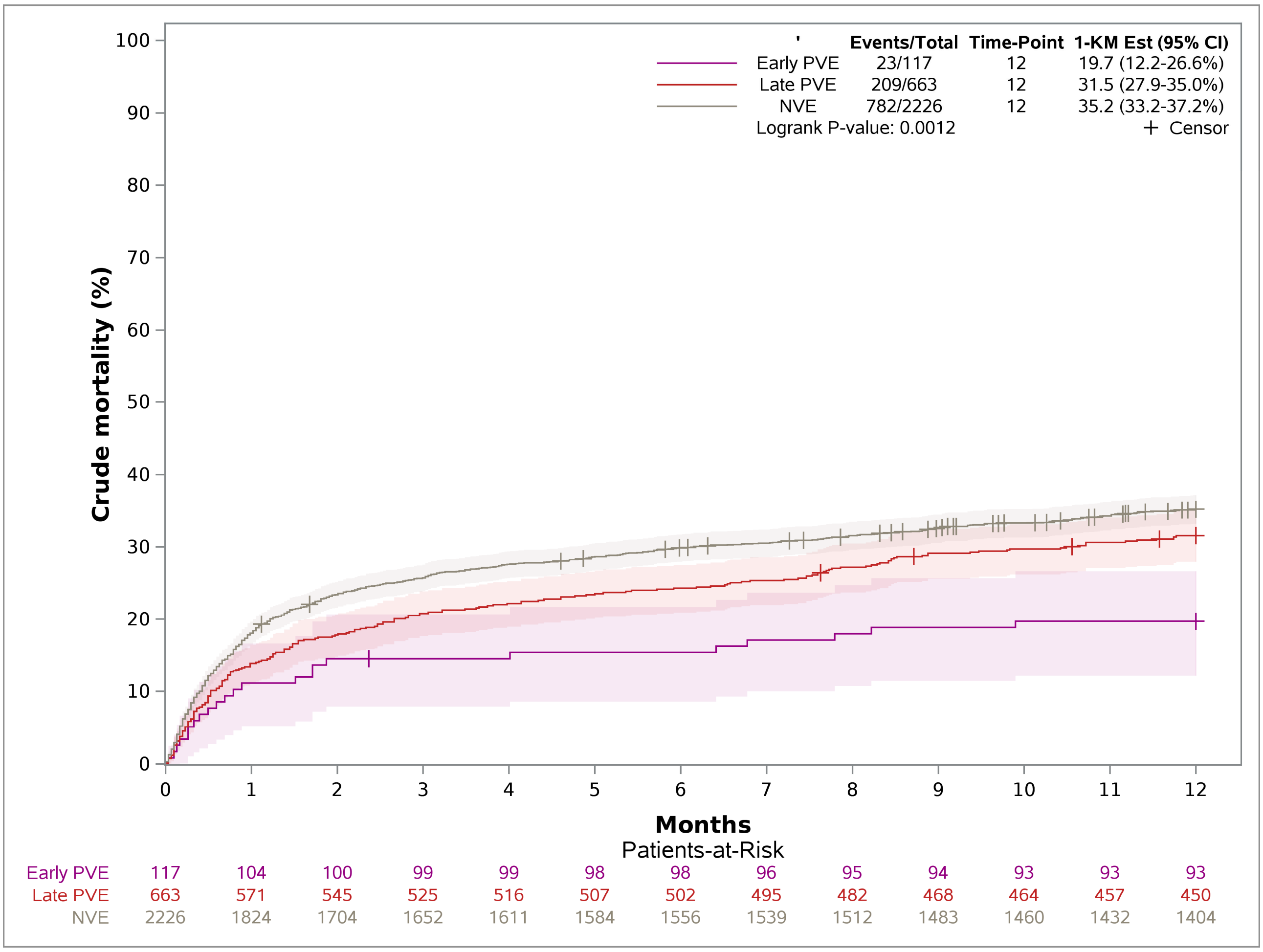

Supplemental Figure S4: Crude one-year mortality (reversed Kaplan-Meier estimator) stratified on early PVE vs. late-PVE defined as defined as ≤ 182 days and >183 days. NVE = native valve endocarditis. PVE = prosthetic valve endocarditis
